# Impact of SARS-CoV-2 on Viral Respiratory Infections in Patients with Hematological Malignancies

**DOI:** 10.3390/v16101520

**Published:** 2024-09-26

**Authors:** Antonio Giordano, Martina Quattrone, Marcello Viscovo, Barbara Fiori, Rosaria Santangelo, Maurizio Sanguinetti, Livio Pagano

**Affiliations:** 1 Dipartimento Scienze di Laboratorio ed Ematologiche, Fondazione Policlinico Universitario A. Gemelli, IRCCS, 8 I-00168 Roma, Italy; martyq211@gmail.com (M.Q.); viscovomarcello@gmail.com (M.V.); barbara.fiori@policlinicogemelli.it (B.F.); rosaria.santangelo@policlinicogemelli.it (R.S.); maurizio.sanguinetti@unicatt.it (M.S.); livio.pagano@unicatt.it (L.P.); 2 Hematology Department, Fondazione Policlinico Universitario A. Gemelli, IRCCS, Largo A. Gemelli, 8 I-00168 Rome, Italy; 3 Dipartimento di Scienze Biotecnologiche di Base, Cliniche Intensivologiche e Perioperatorie, Università Cattolica del Sacro Cuore, 8 I-00168 Roma, Italy; 4 Dipartimento di Diagnostica per Immagini, Radioterapia Oncologica ed Ematologia, Università Cattolica del Sacro Cuore, 8 I-00168 Roma, Italy

**Keywords:** respiratory infections, virus, COVID-19

## Abstract

Patients with hematological malignancies (HMs) are at high risk of respiratory viral infections due to the intrinsic deterioration of the immune system and chemotherapy treatments. In the recent past, SARS-CoV-2 respiratory viral infection has been responsible for most infectious complications in HMs. We analyzed 2950 samples from 505 patients admitted to the Hematology department from 2019 to 2023. The aim of this study was to determine the epidemiological trend of respiratory viruses in the SARS-CoV-2 era, the characteristics of the patients involved and their outcomes. In our analysis, we found a reduction in non-SARS-CoV-2 respiratory viral (NSRV) positivity during the pandemic period, although these data did not show statistical significance. Most of the HMs involved were Multiple Myeloma and Acute Myeloid Leukemia. Overall mortality rate was very low and characterized by the progression of the HMs as well as the worsening of respiratory failure. In conclusion, a reduction in non-COVID viral infections was highlighted, probably also thanks to the increase in prevention measures and environmental modifications of the viral background.

## 1. Introduction

Coronaviruses are a heterogeneous group of viruses that can infect different animal species and can cause mild to severe respiratory infections in humans [1]. In 2002 and 2012, respectively, two highly pathogenic Coronaviruses of zoonotic origin, Severe acute respiratory syndrome Coronavirus (SARS-CoV) and Middle East respiratory syndrome Coronavirus (MERS-CoV), were isolated in humans and caused fatal respiratory diseases. Coronavirus disease 2019 (COVID-19), an infectious disease caused by Severe acute respiratory syndrome Coronavirus 2 (SARS-CoV-2), emerged in late 2019 in Wuhan (China) and was declared a global pandemic on 11 March 2020 [1,2]. The phenotypic spectrum of the disease ranges from asymptomatic individuals to patients requiring ventilation due to pneumonia [3]. In severe SARS-CoV-2 infection, genetic background is likely to be of utmost importance in assessing susceptibility to the virus, and the discovery of genetic polymorphisms could be beneficial in the treatment and prevention of infections [4].

Cancer patients are more susceptible to infections than healthy individuals due to the systemic immunosuppressive state generated by chemotherapy [5]. In fact, many patients present a B-cell immune dysfunction with reduced immunoglobulin production, and also, the T cell arm of immunity is dysfunctional in patients with cancer and under chemotherapy; therefore, they are more vulnerable to viral and other infections [6].

In December 2020, vaccines against SARS-CoV-2 became available, and they contributed to the reduction in infection mortality in patients with hematological malignancies (HMs) from 31.2% to 9.2%, also with a reduction in the incidence of severe respiratory syndromes and hospitalization and with improved survival in patients who had received at least the first two doses of vaccine, that initially were dose standard to be considered fully vaccinated [7,8,9]. Even in the vaccinated patient population with HMs, prognosis remains worse in those with advanced age, multiple comorbidities and active hematologic disease [9].

Viral respiratory infections have always been a leading cause of complications and mortality in the general population as well as in patients with HMs [10]. Prior to the SARS-CoV-2 pandemic, Respiratory Syncytial Virus was considered the main virus responsible for viral respiratory infections in the HMs, with increased lower airway involvement and mortality rates up to 43% in some case series [10]. The most frequently isolated non-SARS-CoV-2 respiratory viruses (NSRVs) constitute a heterogeneous group and are mainly RNA viruses: Respiratory Syncytial Virus (RSV), Influenza virus (IV), Parainfluenza virus (HPIV), Metapneumovirus, Rhinovirus and Coronavirus; however, it is also common to detect DeoxyriboNucleic Acid (DNA) viruses such as adenovirus, bocavirus and polyomaviruses. In most cases, however, the etiological agent is not identified due to poorly usable tests and generally self-limiting clinical conditions [11]. Respiratory virus infections frequently have seasonality, with a peak incidence in late fall and winter [12].

Clinical manifestations depend on the type of virus and the patient’s degree of immunosuppression, from asymptomatic infection to severe respiratory distress. In some cases, in addition to respiratory symptoms, diarrhea, conjunctivitis, headache or other systemic symptoms can be found [13]. Often, at onset, there may be nonspecific symptoms with initial upper airway involvement, such as cough, rhinorrhea, otitis and pharyngodynia [13]. Unfortunately, in the specific setting of the HMs, respiratory viruses can also cause hospitalization [14]. Clinical symptoms at the onset of infection do not always help distinguish which patients will progress to a severe form, and diagnostic tests often remain positive for prolonged periods of time, proving insufficient to guide therapeutic intervention [12]. Currently, the real-time polymerase chain reaction (PCR) remains the most used tool [13].

Treatment in patients with HMs and NSRV infection is indeed primarily supportive and includes oxygen for hypoxia, fluid therapy and, when necessary, intensive-care support such as mechanical ventilation [14]. Immunoglobulin replacement and antiviral therapy may be useful in some cases, whereas inappropriate use of antibiotics can generate deleterious selective pressure with the acquisition of drug resistance [13,14].

Several studies have compared the epidemiological and clinical features of SARS-CoV-2 infection with those of NSRV infection, particularly Influenza virus and the 2009 epidemic [15]. Specifically, SARS-CoV-2 showed a higher severity of infection and incidence of mortality than Influenza in the general population but a comparable rate of severe forms (i.e., requiring intensive care) in the hospitalized patient population; however, data are lacking as far as hematologic patients are concerned [15,16].

Our study analyzes more widespread non-SARS-CoV-2 respiratory virus infections (RSV, IV, HPIV) in patients with Hematologic Neoplasms and compares the epidemiology of these infections before and after the COVID outbreak.

## 2. Materials and Methods

### 2.1. Study Population

We analyzed biological samples from patients admitted to the Hematology department of Policlinico Universitario A. Gemelli-IRCCS in Rome (Italy) from January 2019 to December 2023.

All patients who underwent at least one more or less intensive chemotherapy treatment were included in the study, while we excluded patients treated with immunosuppressive therapy or other for non-oncological-hematological disease.

### 2.2. Sample Collection and Testing

In all examined patients, we tested NSRV samples with the Multiplex RT-PCR test with nasal or pharyngeal swabs, sputum, nasal secretion, alveolar broncho-lavages and pleural fluid. Samples were examined as a routine investigation in all febrile events or in patients with respiratory symptoms. The microbiological tests involved the search for major respiratory viruses. We focused our analysis on Respiratory Syncytial Virus (RVS), Influenza virus (IV) and Parainfluenza viruses (HPIVs).

For each patient, we reported the date of positivity of the microbiological test, the hematological neoplasm that was the reason for admission at the time of the onset of the respiratory infection and the clinical outcome parameters, particularly the need for admission to intensive care, the course of the infection 30 days after diagnosis and mortality. Our analysis considered the statistical significance regarding the change in incidence for NSRV positivity using the Chi-Square test.

## 3. Results

**Figure 1:**
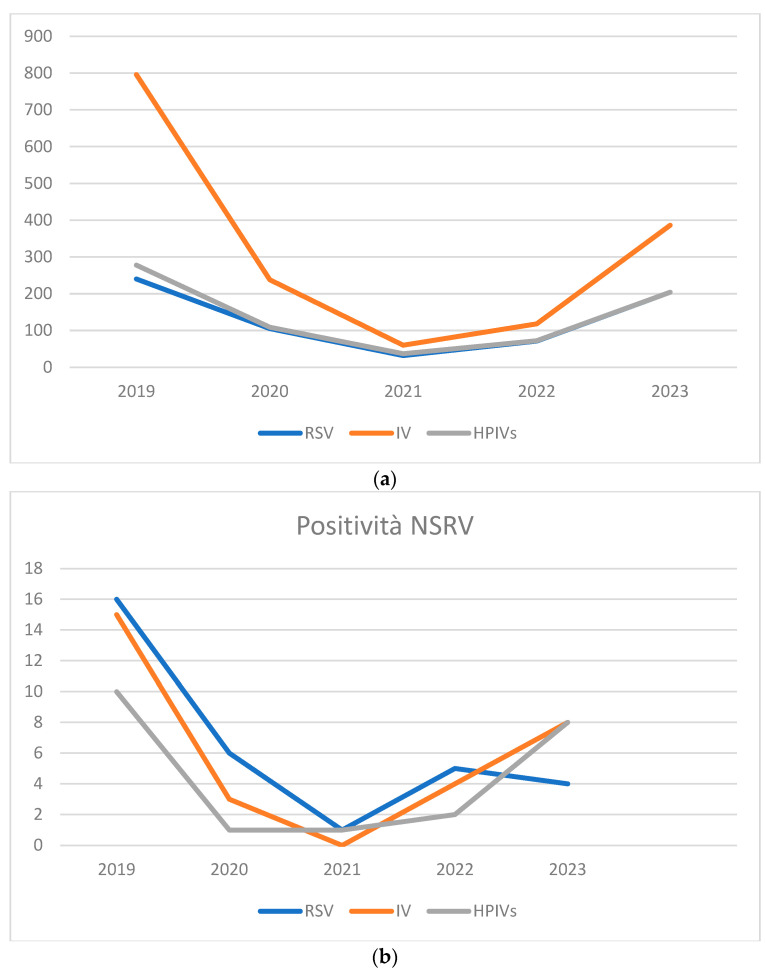
(**a**) Samples analyzed from 2019 to 2023. (**b**) Positive tests for NSRV from 2019 to 2023.

We analyzed 2950 samples from 505 patients admitted to the Hematology department from January 2019 to December 2023 [Table 1]. We tested 652 samples (22.1%) for RSV, 1598 (54.2%) for IV and 700 (23.7%) for HPIVs [Figure 1a]. A total of 84 samples tested positive (2.9%): 32 samples tested positive for RSV (4.9%), 30 (1.9%) for IV and 22 (3.1%) for HPIVs [Figure 1b].

Overall, 76 patients (15.1%) tested positive for NSRV: 32 (7.5%) had RSV, 30 (6.1%) had IV and 22 (4.9%) HPIVs. Moreover, 5 patients tested positive for more than one NSRV; none of these required intensive care, and all resolved the respiratory complication, although only in one case was there an improvement with chronic lung damage.

**Figure 2:**
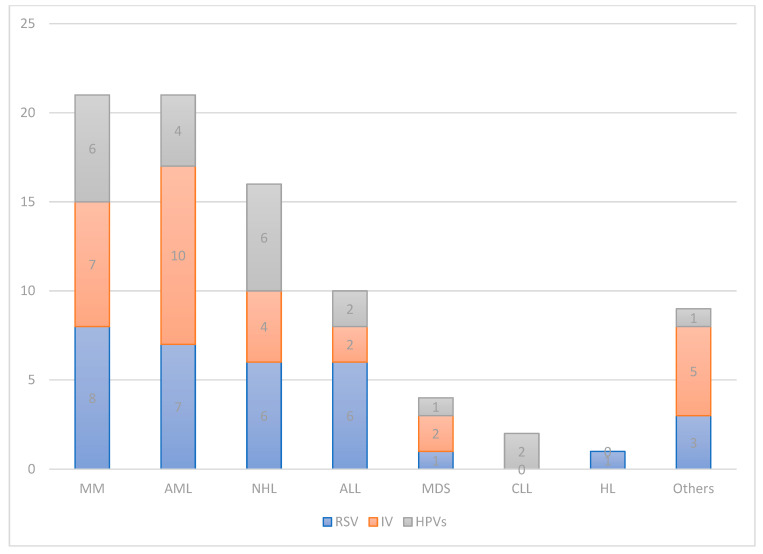
Distribution of infectious events by hematologic malignancy.

As for the HMs by which NSRV-infected patients were affected, the highest number of positives were found in those with Multiple Myeloma (MM, 21 cases, 25%) and Acute Myeloid Leukemia (AML, 21 cases). Sixteen infections occurred in patients with Non-Hodgkin’s Lymphoma (NHL, 19%), 10 in patients with Acute Lymphoblastic Leukemia (ALL, 12%), 4 in Myelodysplastic Syndromes (MDS, 5%) and 2 in Chronic Lymphatic Leukemia (CLL, 2%) [Table 2, Figure 2].

Stratifying our results by year, in 2019, we collected 1314 samples for NSRV, specifically 240 for RSV (18.3%), 796 for Influenza virus (60.6%) and 278 (21.1%) for Parainfluenza [Table 1]. Among these, 41 samples (3.1%) were positive for NSRV, including 16 samples (39.0%) for RSV, 15 (36.6%) for Influenza virus and 10 (24.4%) for Parainfluenza. In 2020, 452 microbiological samples for NSRV were tested, including 105 for RSV (23.2%), 238 for Influenza virus (52.7%) and 109 for Parainfluenza. Of them, 10 tests were positive (2.2%), including 6 positive samples for RSV (60%), 3 for Influenza virus (30%) and 1 for Parainfluenza (10%).

In 2021, 129 samples were tested for NSRV, including 32 for RSV (24.8%), 60 for Influenza virus (46.5%) and 37 for Parainfluenza (28.7%). Of these, 2 samples were positive (1.6%): 1 for RSV and 1 for Parainfluenza. In 2022, 261 requests were examined for NSRV, including 71 for RSV (27.2%), 118 for Influenza virus (45.2%) and 72 for Parainfluenza (27.6%). There were 11 (4.2%) samples that tested positive, including 5 for RSV (45.5%), 4 for Influenza virus (36.4%) and 2 for Parainfluenza (18.2%).

Finally, in 2023, we analyzed 794 samples for NSRV, including 204 for RSV (25.7%), 386 for Influenza virus (48.6%) and 204 for Parainfluenza (25.7%). There were 20 positives (2.5%), including 4 for RSV (20%), 8 for Influenza virus (40%) and 8 for Parainfluenza (40%).

We also analyzed the clinical outcome of NSRV infection in our cohort of hematological patients. Among the 76 positive patients, 5 (6.6%) were admitted to the Intensive Care Unit (ICU). Two of them had AML, 1 with MM, 1with NHL, and 1 was affected by Chronic Prolymphocytic Leukemia.

Thirty days after the infection, 64 (84.2%) patients had a complete resolution of their symptoms, 7 (9.2%) had only a clinical improvement, 3 (4.0%) experimented a deterioration of their clinical conditions and 2 (2.6%) died. The causes of death were progression of the hematological disease and worsening of respiratory function in both cases.

Among the 76 patients who tested positive, 32 (42.1%) died during the observation period: 29 (90.6%) had disease progression, and 3 died from septic shock due to bacterial infections.

## 4. Discussion

Lung infections have been for many years, and still are, the main cause of infectious mortality in HM patients. The main agents of these infections have basically been identified in molds (e.g., Aspergillus, Mucorales) and Gram-bacteria (e.g., Pseudomonas aeruginosa, Klebsiella pneumoniae) [17,18,19]. These germs are responsible for high mortality and morbidity in both patients undergoing chemotherapy and those undergoing HSCT. The viral agents responsible for respiratory infections have been underestimated for years by many clinicians, probably due to the lack of therapies considered effective, but the recent COVID-19 pandemic has reawakened attention for this type of infectious complications with a timely diagnosis and possible treatment.

At present, there are no substantial data that allow us to know the incidence rate of viral respiratory infections in the various categories of hematological patients with different hematological malignancies and the impact that these may have on the outcome, apart from transplant patients where a flu mortality rate of 6% has been reported [20].

Respiratory viral infections have always been a prerogative of the HMs and are therefore part of the usual management of the febrile event. In recent years, in fact, many case series have demonstrated how respiratory viral infections can be the cause in this context of severe pneumonia up to “Acute Respiratory Distress Syndrome” [21]; in most cases, the data were limited to specific contexts, i.e., patients undergoing HSCT with attributed mortality rates up to 43% [14]. Other studies have instead analyzed the trend in specific infections such as RSV, considered the first cause of respiratory viral infection, especially in HSCT recipients, with incidence rates up to 30% and subsequent hospitalization for pneumonia [10]. The environmental pressure of the SARS-CoV-2 infection appears to have modified the incidence of other respiratory viruses, reducing and even eliminating clinically evident infections such as Influenza and RSV.

Some data to support this theory have demonstrated that, during the pandemic period, there was a reduction in the incidence of viral pulmonary etiology for NSRVs from approximately 30% up to 6%, while the incidence of bacterial and fungal etiologies remained stationary [22].

The use of containment measures (use of masks, distancing and hand washing) has probably contributed to the clear reduction in all transmitted respiratory infections, which are generally more virulent. This hypothesis cannot be proven at the moment. It is also useful to consider that 27 December 2020, the so-called “Vaccine day”, is the date that marked the official start of the vaccination campaign against COVID-19 throughout Europe. In Italy, the actual distribution of the vaccine began on 31 December 2020.

This study confirms the now-consolidated need to use preventive measures in the population of patients affected by hematological malignancies.

## 5. Conclusions

In our analysis, the reduction in the positivity rate was not statistically significant, although these data were affected by a global reduction in swabs performed for non-COVID respiratory viruses due to the ongoing pandemic.

Furthermore, these data have allowed us to understand the epidemiological distribution of the respiratory viruses most frequently responsible for infections in HMs. These complications, although reduced in the period of greatest diffusion of SARS-CoV-2, are still considered a constant infectious risk with a significant healthcare burden in terms of hospitalization.

The data we have collected indicate that we should not neglect the search for these infections in the management of febrile events in patients with HMs. Furthermore, future studies will be necessary to establish how the epidemiological impact of the viruses examined can be decisive in the context of concomitant pulmonary infections caused by bacteria or fungi and how this influences the outcomes.

## Figures and Tables

**Table 1:** Samples analyzed for RSV, Influenza virus and Parainfluenza from 2019 to 2023.

	2019	2020	2021	2022	2023	Total	*p*
**RSV**	16/240 (6.7%)	6/105 (5.7%)	1/32 (3.1%)	5/71 (7.0%)	4/204 (2.0%)	32/652 (4.9%)	0.17
**Influenza Virus**	15/796 (1.9%)	3/238 (1.3%)	0/60	4/118 (3.4%)	8/386 (2.1%)	30/1598 (1.9%)	0.74
**Parainfluenza**	10/278 (3.6%)	1/109 (0.9%)	1/37 (2.7%)	2/72 (2.8%)	8/204 (3.9%)	22/700 (3.1%)	0.73
**Total**	41/1314 (3.1%)	10/452 (2.2%)	2/129 (1.6%)	11/261 (4.2%)	20/794 (2.5%)	84/2950 (2.9%)	0.4

**Table 2:** Distribution of infectious events by hematologic malignancy.

Hematologic Disease	RSV	IV	HPIVs	Total
**MM**	8	7	6	21
**AML**	7	10	4	21
**NHL**	6	4	6	16
**ALL**	6	2	2	10
**MDS**	1	2	1	4
**CLL**	0	0	2	2
**HL**	1	0	0	1
**Others**	3	5	1	9

## Data Availability

The original contributions presented in the study are included in the article, further inquiries can be directed to the corresponding author/s.
